# Adenine Nucleotide Translocase, Mitochondrial Stress, and Degenerative Cell Death

**DOI:** 10.1155/2013/146860

**Published:** 2013-07-18

**Authors:** Yaxin Liu, Xin Jie Chen

**Affiliations:** Department of Biochemistry and Molecular Biology, State University of New York Upstate Medical University, Syracuse, NY 13210, USA

## Abstract

Mitochondria are intracellular organelles involved in ATP synthesis, apoptosis, calcium signaling, metabolism, and the synthesis of critical metabolic cofactors. Mitochondrial dysfunction is associated with age-related degenerative diseases. How mitochondrial dysfunction causes cell degeneration is not well understood. Recent studies have shown that mutations in the adenine nucleotide translocase (Ant) cause aging-dependent degenerative cell death (DCD) in yeast, which is sequentially manifested by inner membrane stress, mitochondrial DNA (mtDNA) loss, and progressive loss of cell viability. Ant is an abundant protein primarily involved in ADP/ATP exchange across the mitochondrial inner membrane. It also mediates basal proton leak and regulates the mitochondrial permeability transition pore. Missense mutations in the human Ant1 cause several degenerative diseases which are commonly manifested by fractional mtDNA deletions. Multiple models have been proposed to explain the Ant1-induced pathogenesis. Studies from yeast have suggested that in addition to altered nucleotide transport properties, the mutant proteins cause a global stress on the inner membrane. The mutant proteins likely interfere with general mitochondrial biogenesis in a dominant-negative manner, which secondarily destabilizes mtDNA. More recent work revealed that the Ant-induced DCD is suppressed by reduced cytosolic protein synthesis. This finding suggests a proteostatic crosstalk between mitochondria and the cytosol, which may play an important role for cell survival during aging.

## 1. Introduction

Mitochondria are essential organelles as they produce most of ATP to support cellular activities, synthesize critical metabolic factors such as heme and iron-sulfur clusters, and are involved in lipid and phospholipid metabolism as well as calcium signalling [1]. Mitochondria also play an important role in determining the fate of cell via their involvement in cell death. Cell death can be classified into different categories. According to the morphological appearance, for instance, cells undergo death via necrosis (accidental cell death or programmed necrosis), apoptosis, or aberrant autophagy, all with significant involvement of mitochondria. In yeast, mitochondria-mediated apoptosis is believed to execute with some steps common to the mammalian cells. Oxidative burst, mitochondrial fragmentation, the collapse of mitochondrial membrane potential, and the release of cytochrome *c* are commonly observed in apoptotic yeast cells [2, 3].

In addition to the relatively acute forms of cell death aforementioned, mitochondrial function also progressively deteriorates during aging, which leads to cellular senescence. It is conventionally thought that mitochondria contribute to aging mainly through the overproduction of reactive oxygen species (ROS) and underproduction of ATP in aged cells. Interestingly, recent studies have suggested that mitochondria may overcome these stresses and promote cell survival by altered cellular signalling [4–7]. In this review, we will present a novel form of mitochondria-induced cell death in yeast cells, tentatively referred as degenerative cell death (DCD). DCD is characterized by mitochondrial inner membrane stress, mtDNA damage, and progressive loss of cell viability. The key feature of DCD, which is distinct from the currently known forms of cell death in yeast (e.g., apoptosis and necrosis), is the loss of mtDNA which cannot be tolerated by cells with compromised inner membrane integrity. This was revealed by studying some mutant forms of adenine nucleotide translocase, which causes aging-dependent cellular degeneration. These studies may provide new perspectives for the mechanism of mitochondrial degeneration, in addition to the well-established roles of oxidative stress and mitochondrial quality control which contribute to aging. 

## 2. Physiological Roles of Adenine Nucleotide Translocase

Adenine nucleotide translocase (or adenine nucleotide translocator or Ant) is the most abundant protein in mitochondria, accounting for up to 10% of total mitochondrial protein content [8]. It is encoded by the nuclear DNA, synthesized in cytosol, imported into mitochondria, and finally inserted into the inner membrane [9–11]. Ant belongs to the mitochondrial carrier family (MCF) proteins, a class of proteins that plays an important role in the transport of metabolites and cofactors across the mitochondrial inner membrane [12, 13]. The primary function of Ant is to catalyze ADP/ATP exchange across the inner membrane. Under respiring conditions, ATP^4−^ generated by oxidative phosphorylation is exported to the cytosol for use in cellular activities and ADP^3−^ is imported into the mitochondrial matrix for continuous ATP synthesis. Ant is therefore an ADP^3−^/ATP^4−^ exchanger. During this strict exchange process, one net negative charge is moved from the matrix to the cytosol, resulting in a charge differential that is driven by membrane potential across the mitochondrial inner membrane [14]. Ant binds to its substrates with relatively low affinity, while its high abundance can compensate for the inefficient transport. Ant also has an intrinsic property of mediating proton leakage [8]. In addition to its involvement in a fatty acid-dependent proton leakage pathway, it accounts for 1/2 to 2/3 of the basal proton conductance through an unknown mechanism. Hence, Ant can result in mild uncoupling and decrease efficiency of ATP synthesis. 

The Ant protein has multiple isoforms in different species. In humans, there are four isoforms that have distinct tissue-specific expression patterns. Ant1 is predominantly expressed in postdifferentiated tissues such as heart and skeletal muscle [15]. Ant2 is more abundant in certain proliferating tissues [16, 17]. Ant3 is ubiquitously expressed and Ant4 is specifically expressed in the testis [18]. Only three isoforms of Ant have been found in mouse (Ant1, 2, and 4). Mouse Ant1 is a heart/skeletal muscle specific isoform, while mouse Ant2 is highly expressed in all tissues except muscle. Mouse Ant4 is expressed primarily in testis as in humans [15, 18]. Yeast contains three isoforms of ADP/ATP carrier (Aac), which are homologues of Ant in humans. Aac2 is the major ADP/ATP carrier in aerobically grown yeast cells [19]. 

**Figure 1:**
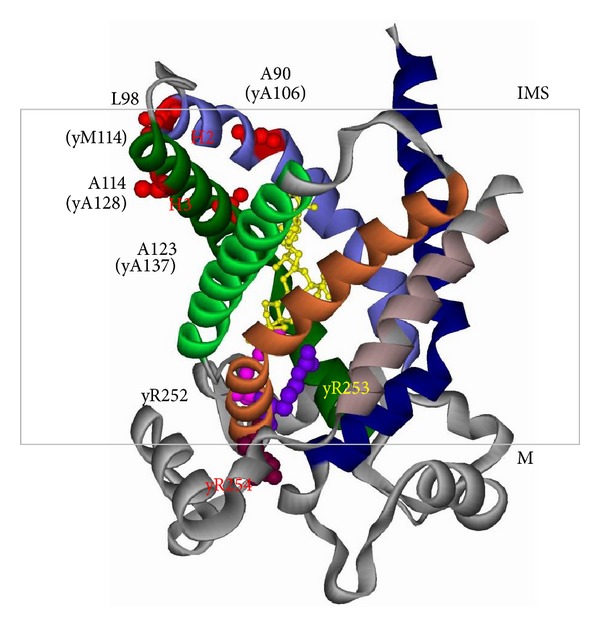
Projected localization of A90, L98, A114, A123, and the arginine triplet on the crystal structure of bovine Ant1 in the cytosolic conformation bound by CATR (yellow) [25]. The corresponding amino acids in yeast Aac2 are also indicated. R252, R253, and R254 in yeast correspond to R234, R235, and R236 in the bovine protein. IMS, intermembrane space; M, matrix.

Like other members in MCF, Ant has three repeats of a ~100 amino acid sequence and each repeat contains two transmembrane domains that form alpha helices [20, 21]. Biochemical characterization of Ant benefited from two specific inhibitors of Ant, bongkrekic acid (BA) and carboxyatractyloside (CATR). Both of the inhibitors bind with the stoichiometry of one inhibitor per two molecules. BA binds the matrix side of Ant and CATR binds on the intermembrane space side [22–24]. The transition between CATR and BA conformations is suggested to be the structural switch involved in ADP/ATP transport [19]. The crystal structure of bovine Ant complexed with CATR revealed the organization of six transmembrane domains with both N- and C-termini extending into the intermembrane space [25] (Figure 1). All the transmembrane domains are *α*-helices, which are tilted and form a cavity with the opening toward the intermembrane space. Three kinks are introduced by proline residues between two helices on the matrix side of Ant, which may act as hinges to facilitate the opening and closing of the nucleotide translocation channel [25]. Recent study of yeast Aac2 using hydrogen/deuterium exchange-mass spectrometry showed that the BA-bound Aac2 is structurally different from the CATR-bound form. The BA conformation has better solvent accessibility from the matrix side [26, 27]. Ant has the RRRMMM signature sequence, which is absent from other mitochondrial carriers. This motif spans over the thinnest part of the channel and the arginine residues are essential for attracting the negatively charged nucleotides to facilitate transport [25, 28, 29]. In addition to the arginine residues, the methionine triplet also contributes to nucleotide translocation or binding [27]. 

It has been debated whether Ant functions as a monomer or a dimer. A dimeric structure was first suggested by native gel electrophoresis, ultracentrifugation, neutron scattering, and cross-linking studies [30–35]. In the dimer model, the C terminus of one monomer is predicted to be close to the N terminus of a second monomer [36–38]. However, more recent reports suggested that Ant may be present in a monomeric form. The crystal structure of bovine Ant1 was solved as a monomer [25]. The study using differential tagging showed that the yeast Aac2 is a monomer in mild detergents because tagged Aac2 does not form dimers with untagged Aac2 [39]. Other techniques such as analytical ultracentrifugation, small-angle neutron scattering and electron cryomicroscopy also suggested that Ant more likely functions as monomers (reviewed in [40]). 

Ant may also affect the mitochondrial permeability transition pore (mPTP) on the inner membrane, but its exact role in this activity has been highly debated [41–44]. Elevated Ca^2+^ and other factors are involved in the stimulation of mPTP opening followed by increased permeability of solutes across the inner membrane, which results in the dissipation of membrane potential, mitochondrial swelling, and finally cell death through apoptosis or necrosis. It has also been documented that mPTP plays a role in mediating organismal aging [45]. Early studies suggested Ant as a critical component of mPTP, along with voltage-dependent anion channel (VDAC) in the outer membrane and cyclophilin D (CyPD) in the matrix [46, 47]. However, Kokoszka et al. inactivated the two Ant isoforms in mouse and still detected the opening of mPTP triggered by Ca^2+^, suggesting that Ant is not essential for mPTP [48]. Ant still plays a role in the regulation of mPTP since more Ca^2+^ are required to activate the mPTP and the Ant ligands no longer regulate the mPTP. The very recent studies defined the mPTP as the dimers of the F_*o*_F_1_-ATP synthase that is regulated by CyPD [49] and the c subunit of the enzyme appears to be critical for permeability transition [50]. Given that adenine nucleotides are the substrates of the ATP synthase, Ant may contribute to mPTP regulation by affecting nucleotide levels in the matrix where the F_1_-ATPase sector of the ATP synthase is located. It has been reported in yeast that loss of Ant (or Aac) protects cells from acetic acid and diamide-induced mitochondrial outer membrane permeabilization, mitochondrial degradation, and apoptosis [51–53]. Interestingly, this was proposed to involve mPTP probably via an activity independent of nucleotide translocation.

## 3. Altered Ant Expression and Human Diseases

Given the importance of Ant to mitochondrial physiology, mutations or altered expression of Ant has been found to be associated with a growing list of human diseases (Table 1). In all the four Ant isoforms, Ant1 is so far the only one found to directly cause mitochondrial diseases. Early work has shown that *ANT1* ^−/−^ mice presented overproliferation of mitochondria in skeletal and heart muscles, ragged-red fibers (fibers that have a ragged contour and an accumulation of red staining material which is associated with proliferation of abnormal mitochondria), cardiac hypertrophy, exercise intolerance, lactic acidosis, and deficiency in coupled respiration in mitochondria [54]. In humans, deficiency in Ant1 is associated with Senger's syndrome, an autosomal recessive disease characterized by hypertrophic cardiomyopathy, mitochondrial myopathy, lactic acidosis, and congenital cataracts [55]. Although depletion of Ant1 in heart and muscle tissues has been proposed to be the primary cause of the Senger's syndrome, no mutations have been found in *ANT1*. It has been speculated that the transcription, translation, or posttranslational modification of Ant1 may be affected [56]. Recently, two nonsense mutations in the gene encoding the mitochondrial acylglycerol kinase (AGK) were identified from a patient with typical symptoms of Senger's syndrome [57]. AGK is a multisubstrate lipid kinase involved in phospholipid metabolism. The loss of AGK may result in the decrease of Ant by affecting its biogenesis. In addition to Senger's syndrome, loss of Ant1 due to a homozygous null mutation also causes cardiomyopathy, and the severity of the cardiac disease correlates with the mtDNA haplogroup. Patients with the haplogroup U mtDNAs are more affected than those having the haplogroup H [58]. Overall, deficiency in Ant1 expression and biogenesis would be expected to cause not only reduced ATP output but also oxidative damage because of F_*o*_F_1_-ATP synthase stalling, increased electron leak, and ROS production.

In contrast to Ant1 deficiency, overexpressed Ant1 may contribute to the pathogenesis of other diseases such as facioscapulohumeral muscular dystrophy (FSHD). FSHD is a highly variable autosomal dominant neuromuscular disorder. Patients with FSHD suffer from cumulative progression of muscle weakness in the face, feet, shoulders, and hips, along with occasionally sensorineural hearing loss [59]. Deletions of the D4Z4 repeated sequences on chromosome IV are commonly found in FSHD patients, which may lead to transcriptional derepression of nearby genes including *ANT1*, *FRG1*, *FRG2*, and *DUX4 *[60–63]. Overexpression of *FRG1*, a gene involved in pre-mRNA splicing, and not *ANT1*, was proposed to be responsible for FSHD [64]. Other studies instead proposed that expression of *DUX4* is critical for FSHD pathogenesis [63]. Nevertheless, recent studies have also reported the overexpression of Ant1 and increased oxidative stress in FSHD muscles [65]. These observations suggest that Ant1 may play a role in the pathogenesis of FSHD. 

Although the sequence identity between the Ant isoforms is as high as 70%~90% [66], their nucleotide transport properties may differ and altered expression of these isoforms could have different metabolic consequences. For example, *ANT2* is upregulated in hormone-dependent cancers. Brenner et al. showed that *ANT2* mRNA is significantly elevated in primary tissues derived from patients with breast, uterus, ovary, lung, thyroid gland bladder, and testis cancers [67]. Unlike healthy cells, cancer cells intensively employ glycolysis and have reduced oxidative phosphorylation (OXPHOS) to adapt to the intratumoral hypoxic conditions [68]. It has been known in yeast that when cells are severely compromised in mitochondrial function (e.g. *ρ* ^*o*^ cells), the mitochondrial membrane potential is maintained through reversed nucleotide transport by the ADP/ATP carrier. ATP is imported into the matrix, where it is recycled back to ADP as long as an active F_1_-ATPase is present. ADP is then exported into the cytosol. This reversal ADP/ATP exchange is critical for mitochondrial biogenesis and cell viability under severe mitochondrial damage conditions [69–72]. By analogy, cancer cells are speculated to import glycolytically produced ATP into mitochondrial matrix via Ant2 [73]. The human F_*o*_F_1_-ATPase may hydrolyze ATP to ADP that facilitates the electrogenic ATP_cystol_^4−^/ADP_matrix_^3−^ exchange [74]. Given the key role of Ant2 in cancer metabolism, it may be used as a potential target for cancer therapy. 

## 4. Dominant Mutations in Ant1 and Human Diseases

Missense mutations in Ant1 have been found to cause several human diseases. One of them is autosomal dominant Progressive External Ophthalmoplegia (adPEO), which is characterized by late or adult onset muscle weakness (especially in eye muscles), exercise intolerance, sensory ataxia, hypertrophic cardiomyopathy, and myopathy [75–77]. Multiple mtDNA deletions and mild defects in the respiratory complexes were detected in affected tissues. adPEO is also caused by specific missense mutations in the mitochondrial twinkle helicase or in the mtDNA specific polymerase, Pol*γ* [78, 79], which are directly involved in mtDNA replication. Moreover, a total of five Ant1 missense mutations have been reported in one sporadic and four familial cases of adPEO. Most of these mutations occur in highly conserved amino acids: Ala90, Leu98, Asp104, Ala114 and Val289 [75–77, 80]. In addition to adPEO, the A123D missense mutation has been identified in a homozygous patient, which is manifested by slow progressive mitochondrial myopathy and cardiomyopathy, but not opthalmoplegia [81]. All those six mutable amino acids except Val289 locate in the helix 2-loop-helix 3 region (Figure 1), which is suggested to undergo dynamic structural changes during nucleotide transport [82]. A later study has shown that the sporadic V289M mutation is accompanied with a mutation in *POLG1*, the gene encoding the large subunit of Pol*γ* [83]. Thus, the contribution of this particular Ant1 mutant allele to the pathogenesis is uncertain when present at a heterozygous state.

## 5. Models for Human Diseases Caused by Gain-of-Function Ant1 Mutations

**Figure 2:**
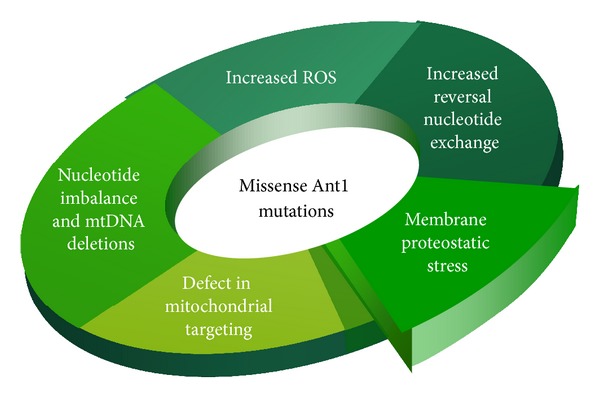
Proposed models for the pathogenic mechanisms of human diseases induced by dominant missense Ant1 mutations. These models predict that the mutant proteins (1) are defective in targeting onto the mitochondrial inner membrane; (2) are defective in nucleotide transport which sequentially causes ATP overaccumulation in the matrix, electron transport chain stalling, membrane hyperpolarization, increased ROS production, and oxidative damage; (3) are engaged in the futile ATP_cytosol_/ATP_matrix_ exchange which leads to matrix nucleotide imbalance and mtDNA deletions; (4) reverse the ADP_cytosol_/ATP_matrix_ exchange under normal conditions which also leads to ATP overaccumulation in the matrix; and (5) cause proteostatic stress on the mitochondrial inner membrane.

 Several model systems have been developed to study the pathogenic mechanism of human diseases caused by the gain-of-function mutations in Ant1 (Figure 2). Kaukonen et al. first introduced the adPEO-type Aac2^*A128P*^ allele, equivalent to human Ant1^*A114P*^, in haploid yeast strains that are disrupted of the *AAC1* and *AAC2* genes [75]. Cells expressing the mutant allele showed a growth defect on nonfermentable carbon sources. The data suggested that the A128P mutation may affect ADP/ATP translocation. This may lead to imbalanced adenine nucleotides, altered intramitochondrial dATP levels in the matrix, and, ultimately, multiple mtDNA deletions.

 In a subsequent study, the human *ANT1* gene was expressed in a yeast mutant disrupted of all the three *AAC* genes [84]. The Ant1^L98P^ and Ant1^V289M^ variants were introduced to evaluate their efficiency in promoting respiratory growth. The complementation test showed that in contrast to the wild type *ANT1*, the two mutant alleles failed to restore cell growth on the nonfermentable lactate medium. Interestingly, the mutant proteins were not detected in the mitochondrial fraction of yeast cells by atractyloside binding and immunodecoration assays, whereas the RNA levels of the mutant *ANT1* were comparable with the wild type. This observation led to the speculation that Ant1^L98P^ and Ant1^V289M^ may not be imported into mitochondria in human diseases.

 As the primary function of Ant is to promote adenine nucleotide transport, the simplest explanation for Ant1-induced adPEO is that the mutant proteins are defective in the transport activity. The dominant phenotypes can only be explained by assuming that Ant1 operates in a dimeric form or that the heterozygous cells are haploinsufficient for the protein. Fontanesi et al. found that the growth of haploid yeast cells expressing only *aac*2^*A*128*P*^, *aac*2^*M*114*P*^, or *aac*2^*S*303*M*^ was severely affected on nonfermentable carbon sources [85]. In addition, cytochrome content, cytochrome *c* oxidase activity, and mitochondrial respiration were all decreased in the mutant cells. In heteroallelic haploid cells, in which the wild type and mutant *aac2* were coexpressed, the level of mitochondrial respiration remained low. This is consistent with the dominant nature of adPEO pathogenesis. The authors also measured the transport properties of Aac2^A128P^, Aac2^M114P^, and Aac2^S303M^ by determining ATP homoexchange rate, ADP homoexchange rate, and ADP/ATP heteroexchange rate in reconstituted proteoliposomes *in vitro*. Interestingly, all the three mutant proteins still retain a robust transport activity for ATP and ADP. However, the mutant proteins preferentially import ATP over ADP. The authors proposed that this may lead to a futile ATP/ATP homoexchange instead of the physiologically productive ATP_matrix_/ADP_cytosol_ heteroexchange mode. This may ultimately result in elevated mitochondrial ATP level. One possible consequence of ATP/ADP imbalance is the increased dATP level, which in turn affects the accuracy of mtDNA replication [85]. However, it is important to note that dATP is likely imported directly from cytosol in yeast rather than converted from ADP or ATP in the mitochondrial matrix. Whether the altered transport properties have physiological implications especially in heterozygous diploid cells needs to be further evaluated. 

 Another model proposed by Kawamata et al. also involves a possible alteration to the nucleotide transport property of Ant1. These authors evaluated the effect of Ant1^A114P^ and Ant1^V289M^ on mitochondrial function in the mouse C2C12 myotube cells [86]. Exogenous Ant1 mutant proteins were confirmed to be localized on the mitochondrial inner membrane. However, no significant differences on oxygen consumption, ATP synthesis, total cellular ATP level, CATR sensitivity, or mtDNA content were detected between the cells expressing the mutant and wild type Ant1. Of interest, mitochondria of A114P-, but not V289M-, expressing myotubes were found to have a reduced ADP/ATP exchange rate and a slightly smaller ADP-induced depolarization. Reduced ADP-induced depolarization suggests a defect in ADP translocation. In addition, cells expressing the A114P and V289M alleles showed abnormal translocator reversal potential. They were switched to the ATP_cytosol_/ADP_matrix_ exchange mode at a higher membrane potential. It was speculated that mutant Ant1 is more prone to invert the direction of ADP/ATP exchange even at the membrane potential still in the physiological range for ATP synthesis. This may lead to increased ATP and nucleotide imbalance in the mitochondrial matrix. Importantly, this phenotype is not caused by loss of function, because the ADP/ATP exchange rate in Ant1-silenced myotubes showed different properties.

Whether altered nucleotide transport is the pathogenic mechanism of Ant1-induced diseases is still inconclusive. The study of Aac2^A137D^ in yeast provided some useful information. Aac2^A137D^ is equivalent to the human Ant1^A123D^ mutation, which does not cause ophthalmoplegia in a homozygous patient but share other common symptoms with adPEO patients including hypertrophic cardiomyopathy, mild myopathy, ragged muscle fibers, exercise intolerance, lactic acidosis, and accumulation of mtDNA deletions. Yeast cells expressing only Aac2^A137D^ are respiratory deficient as they do not grow on nonfermentable carbon sources [81]. The *in vitro* reconstitution assay showed that Aac2^A137D^ completely lacks the ability to transport ATP or ADP. This provides strong evidence that mtDNA deletions in the Ant1^A123D^ patient arise independently of nucleotide transport. The yeast *aac*2^*A*137*D*^ cells have a low viability, which is suppressed by ROS scavengers. This supports the idea that the mutant is vulnerable to oxidative stress and anti-ROS treatments may be a potential therapeutic strategy [81]. 

## 6. The Proteostatic Stress Model

More recent studies in yeast supported the idea that *aac2* alleles resembling the human pathogenic *ant1* mutations may interfere with general mitochondrial biogenesis in a dominant manner [87, 88]. It was shown that yeast cells coexpressing the mutant *aac2* alleles and the wild type *AAC2* exhibit reduced cellular respiration, suggesting that the electron transport chain is severely damaged. In a yeast strain that overexpresses *aac*2^*A*128*P*^, mitochondria showed dramatic depolarization as well as swelling and disintegration of mitochondria. More importantly, when cells expressing only one chromosomally integrated copy of *aac*2^*A*128*P*^ were incubated at 25°C, cell growth is inhibited on glucose medium. Yeast is well known for its ability to grow on fermentable carbon sources without mitochondrial respiration. The growth inhibition strongly suggests that expression of the mutant Aac2 interferes with general mitochondrial biogenesis. Furthermore, when two copies of *aac*2^*A*128*P*^, *aac*2^*M*114*P*^, *aac*2^*A*106*D*^, or *aac*2^*A*137*D*^ were intergraded into the genome, the frequencies of respiratory-deficient petite colonies on glucose medium are greatly increased. This observation recapitulates the mtDNA instability phenotype in human adPEO. Petite frequencies are further increased when cells are grown on raffinose plus galactose medium which stimulates respiration. Concomitantly, cell viability is dramatically reduced. 

Additional phenotypes supported the model that expression of the mutant Aac2 causes general mitochondrial damage [88]. Firstly, the expression of mtDNA-encoded protein, Cox2p, is reduced in the *aac2* mutants. Secondly, yeast cells expressing the four *aac2* mutant alleles are intolerant to *ρ* ^*o*^ condition. The *ρ* ^*o*^-lethality phenotype is an indication of low membrane potential. Cells lose viability when membrane potential is further reduced by the loss of mtDNA. Thirdly, these *aac2* mutants are also hypersensitive to the chemical uncoupler CCCP, consistent with the low membrane potential model. Fourthly, cells coexpressing the mutant *aac2 *and wild type alleles have a diminished respiratory control ratio (RCR), which indicates uncoupled respiration. These data support the model that the mutant Aac2 may cause general stress on the membrane, which leads to defects in respiratory complex biogenesis, membrane uncoupling, loss of ion homeostasis, and the inhibition of cell growth.

Since *aac*2^*A*137*D*^ completely lacks nucleotide transport activity but exhibits similar phenotypes as other mutant *aac2* alleles, these results suggest that mitochondrial damage is independent of ADP/ATP exchange. Further evidence came from the analysis of the double mutants combining *aac*2^*A*128*P*^ with mutations in the Arg252–254 triplet which mitigates adenine transport function [89]. It was found that the arginine mutations barley affects the inhibition of cell growth by the *aac*2^*A*128*P*^ allele. Mitochondrial damage is therefore independent of nucleotide transport. In summary, the data suggested that the mutant Aac2 proteins primarily damage the inner membrane, which consequently affects mitochondrial biogenesis. The loss of mtDNA integrity is likely a consequence of membrane stress. 

The global mitochondrial damage model is supported by another study from El-Khoury and Sainsard-Chanet using the filamentous fungi *Podospora anserina* as a model system [90]. The A114P, L98P, and V289M alleles were introduced into the *P. anserina ANT1* ortholog, *PaANT*. The three mutant strains showed a delayed and reduced rate of germination, a slow vegetative growth rate, and other somatic and sexual defects. In *P. anserina*, lifespan is a good indicator of mtDNA integrity. The three mutant strains were suggested to accumulate mtDNA deletions as they showed dramatically reduced lifespan in certain mating type (*mat* ^−^). Interestingly, short lifespan caused by A114P and L98P mutations, but not V289M, was suppressed by the *rmp1-*2 allele, which is one of the two naturally occurring alleles of *rmp1*. The *rmp1* gene is associated with the timing of death and linked to the *mat* locus tightly. The results indicated that the lifespan in the A114P and L98P mutants is dependent on whether it has the *rmp1-*1 or the *rmp1-*2 allele, but not the mating type. Further studies suggested that premature cell death is independent of mtDNA instability. Mutant strains also exhibited decreased ROS production and mitochondrial inner membrane potential, which could not be suppressed by the *rmp1-*2 allele.

Another important finding in the yeast model is that the missense *aac2* alleles are all synthetically lethal with the disruption of the *YME1* gene, which encodes a chaperone/protease on the inner membrane for degradation of misfolded proteins. This observation strongly suggests that proteostatic stress on the membrane may be responsible for the global mitochondrial damage and the inhibition of cell growth [88]. 

## 7. Ant Mutations Induce Degenerative Cell Death

**Figure 3:**
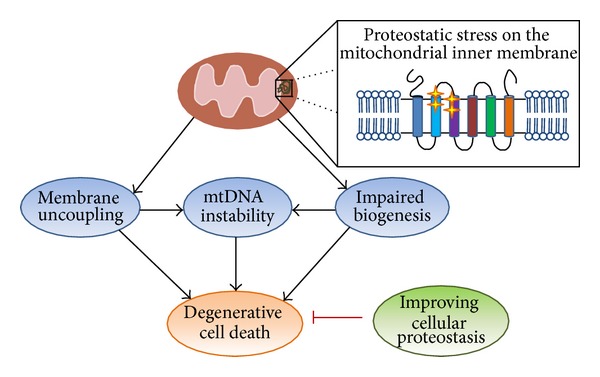
Schematic diagram showing the proteostatic stress model for the pathogenic mechanism of Ant1-induced degenerative cell death. This model predicts that the mutant Ant induces proteostatic stress on the mitochondrial inner membrane, which interferes with general mitochondrial biogenesis and the maintenance of membrane potential, followed by mtDNA destabilization. The severe membrane damage and mtDNA destabilization collectively contribute to degenerative cell death. Degenerative cell death is suppressed by reduced cytosolic protein synthesis which improves global proteostasis. Stars indicate the localization of mutations.

The Chen group found that mitochondrial damage by the mutant *aac2* alleles causes aging-dependent DCD [89]. When yeast cells heterozygous for *AAC*2/*aac*2^*A*128*P*^ were individually spotted on complete glucose medium by micromanipulation, a subfraction of cells formed barely visible microcolonies [87, 89]. The microcolonies contain 2,000~4,000 cells that fail to divide and to produce proliferating lineages. It appears that cells can divide for up to 12~13 generations before complete growth arrest. The delayed loss of the ability to proliferate is termed degenerative cell death (DCD). DCD is likely initiated by membrane stress-induced mtDNA loss (Figure 3). As the *aac2* mutants are *ρ* ^*o*^-lethal, mtDNA loss therefore causes cell death. Cells can continue to divide for limited cell generations after mtDNA loss, probably reflecting either progressive accumulation of cellular factors that inhibit cell division or a dilution of mitochondrial factors that are essential for cell viability. This is supported by pedigree analysis showing that in haploid cells coexpressing *AAC2* and *aac*2^*A*128*P*^, the degenerative mother cell consistently produces degenerative daughter cells. The daughter cells likely inherit the permanently damaged mtDNA from the mother cell which causes cell death. Interestingly, DCD caused by *aac*2^*A*128*P*^ is aging dependent. Replicatively aged mother cells have increased DCD. Approximate half of the founding mother cells have their first degenerative daughter cells after 9~11 cell divisions [89], which is a mid-age onset given that the median lifespan of most *Saccharomyces cerevisiae* strains is about 25 generations.

To understand the mechanism of DCD, much effort has been invested to identify pathways that suppress the degenerative process. It was found that genetic manipulations that reduce cytosolic protein synthesis remarkably suppress DCD [89]. For instance, *RPL6B* encodes a component of 60S ribosomal subunit and its disruption is expected to reduce cytosolic protein synthesis. By meiotic analysis, *aac*2^*A*128*P*^-expressing segregants produce small and sectoring colonies indicative of DCD, whereas cells harboring both *aac*2^*A*128*P*^ and *rpl*6*B*Δ form regular colonies as the wild type. Other genes that suppress in the same manner include *GRP1* (encoding a G-protein-coupled receptor upstream of the protein kinase A pathway), *REI1* (involved in ribosomal biogenesis), *TOR1* and *SCH9* (encoding kinases in the TOR signaling pathway). DCD is also suppressed by cycloheximide, an inhibitor of cytosolic protein synthesis, which further supports the model that reduced cytosolic protein synthesis suppresses mitochondrial degeneration and DCD [89]. In *P. anserina*, premature cell death and mtDNA instability induced by *ANT1* mutations are suppressed by a mutation in the *AS1* gene encoding a ribosomal protein [90].

Expression of the *aac*2^*A*128*P*^ allele shortens the replicative lifespan of yeast cells in a dominant-negative manner, and the shortened lifespan is corrected by the disruption of *RPL6B*, *REI1*, and *SCH9* [89]. This observation supports an epistatic interaction between mitochondrial inner membrane stress and cytosolic protein homeostasis in the control of replicative lifespan. A role of mitochondria in aging and lifespan control is well established [91, 92]. It has been extensively documented that adaptive mitochondria-to-nucleus signaling via the retrograde-response pathway increases cell's lifespan (for review, see [93]). It remains unknown whether *aac*2^*A*128*P*^-induced mitochondrial damage triggers an adaptive adjustment of cytosolic protein homeostasis during replicative aging. 

## 8. Conclusions and Prospects

The pathogenic mechanism of Ant1-induced human diseases is not fully settled. Studies using yeast cells have indicated that the mutant Ant1 may gain a novel property to affect global mitochondrial biogenesis in addition to a potential effect on nucleotide homeostasis. The mutant Ant either directly uncouples the respiration or indirectly compromises protein homeostasis on the membrane which subsequently affects mitochondrial biogenesis. The severe inner membrane damage is manifested by DCD. DCD is initiated by inner membrane damage, followed by mtDNA loss and progressive loss of cell viability because of *ρ* ^*o*^-lethality. These characteristics distinguish it from currently described forms of cell death in yeast including apoptosis and necrosis. Ant-induced DCD is suppressed by reduced cytosolic protein synthesis. This finding strongly suggests that proteostatic stress may play a role in cell degeneration (Figure 3). Future studies are required to understand the mechanism of Ant-induced membrane stress and its contribution to aging-dependent cell degeneration.

## Figures and Tables

**Table 1:** Ant-associated human diseases.

Disease	Mutation	Pathogenic Mechanism	Characteristics
adPEO	*an* *t*1^*A*90*D*^, *ant*1^*L*98*P*^, *ant*1^*D*104*G*^, *ant*1^*A*114*P*^, *ant*1^*V*289*M*^	Membrane stress; altered transport properties	Adult/late-onset, mitochondrial myopathy; muscle weakness (especially in the eyes); sensory ataxia; mtDNA deletions

Cancer	Overexpression of Ant2	Reversed ADP/ATP exchange by Ant2	The adaption to intratumoral hypoxia of cancer cells

FSHD	Deletions of subtelomeric repeats on chromosome IV	*DUX4* overexpression; possibly *ANT1* overexpression	Adult-onset disease, muscle weakness in face, shoulders, and hips, oxidative stress

Mitochondrial myopathy and cardiomyopathy	*ANT1* null mutations	Defect in nucleotide transport	Cardiomyopathy, myopathy, exercise intolerance, and lactic acidosis
	*an* *t1* ^*A*123*D*^	Defect in nucleotide transport; other mechanisms?	

Senger's syndrome	Mutation in AGK affecting Ant biogenesis leads to depletion of Ant1	Defect in nucleotide transport	Cardiac hypertrophy, mitochondrial myopathy, cataracts, lactic acidosis

## Abbreviations

Aac: ADP/ATP carrier
adPEO: Autosomal dominant Progressive External Ophthalmoplegia
AGK: Acylglycerol kinase
Ant: Adenine nucleotide translocase
BA: Bongkrekic acid
CATR: Carboxyatractyloside
CCCP: Carbonyl cyanide *m*-chlorophenyl hydrazone
CyPD: Cyclophilin D
DCD: Degenerative cell death
FSHD: Facioscapulohumeral muscular dystrophy
MCF: Mitochondrial carrier family
mtDNA: Mitochondrial DNA
mPTP: Mitochondrial permeability transition pore
OXPHOS: Oxidative phosphorylation
ROS: Reactive oxidative species
